# Researches into the Physical History of Mankind

**Published:** 1847-04-01

**Authors:** 


RESEARCHES INTO THE PHYSICAL HISTORY OF MANKIND. By *James Cowle Prichard,* M.D., F.R.S., M.R.I.A. Vol. V. 8vo. pp. 570. Sherwood, 1847.

THIS volume, containing the Ethnography of the various people of the innumerable islands of the great Southern Ocean (the Oceanic Nations) and of the American Continent, completes the magnificent work which has been so many years in progress. From the very nature of the *materiel* this portion of the work is less interesting than some of its predecessors; but it is worked out with the same keenness of perception and indefatigable research, bestowed by the author upon them. Having completed his great undertaking, of which our country and profession may well feel proud, Dr. Prichard very modestly, and in our opinion far too briefly, reviews the ground he has passed over. In the first volume he had, from a consideration of the anatomical, physiological, and psychological characteristics of the various races of mankind, announced his conviction that their diversities did not amount to specific differences, but only to mere varieties of one species. The histories of the various races of men inhabiting our globe successively undertaken in these volumes, have furnished a mass of facts corroborative of the analogy deducible from the contemplation of the habitudes of the other portions of animated nature, and converting it into a positive demonstration. At least this is our conviction, and we believe that of most persons who have taken interest in the question since Dr. Prichard commenced its investigation.

